# Data on metals (Zn, Al, Sr, and Co) and metalloid (As) concentration levels of ballast water in commercial ships entering Bushehr port, along the Persian Gulf

**DOI:** 10.1016/j.dib.2016.09.017

**Published:** 2016-09-20

**Authors:** Farshid Soleimani, Sina Dobaradaran, Abdolreza Hayati, Maryam Khorsand, Mozhgan Keshtkar

**Affiliations:** a Department of Environmental Health Engineering, Faculty of Health, Bushehr University of Medical Sciences, Bushehr, Iran; b The Persian Gulf Marine Biotechnology Research Center, The Persian Gulf Biomedical Sciences Research Institute, Bushehr University of Medical Sciences, Bushehr, Iran; c Systems Environmental Health, Oil, Gas and Energy Research Center, The Persian Gulf Biomedical Sciences Research Institute, Bushehr University of Medical Sciences, Bushehr, Iran; d Urban Water and Wastewater Company, Bushehr Province, Iran; e Department of Environmental Engineering Bushehr branch, Islamic Azad University, Bushehr, Iran

**Keywords:** Ballast water, Bushehr, Commercial ships, Metals, Metalloid, Persian Gulf

## Abstract

In this article, we determined the concentration levels of metals including Zn, Al, Sr, and Co and metalloid of As of ballast water in commercial ships entering Bushehr port, along the Persian Gulf. Ballast water samples were taken from commercial ships entering Bushehr port from 34 ports around the world during 15 February and 25 August 2016. The concentration levels of metals and metalloid were determined by using a graphite furnace absorption spectrometer (AAS).

**Specifications Table**

**Table:** 

*Subject area*	*Chemistry*
*More specific subject area*	*Metals and metalloid of ballast water*
*Type of data*	*Table, figure*
*How data was acquired*	*Graphite furnace absorption spectrometer (AAS) method (Varian, SpectrAA 240, Australia).*
*Data format*	*Raw, analyzed*
*Experimental factors*	*Each sample was collected in a 100* *ml sterile container, placed in a cooler at −4* *° C, and transported to the laboratory in the same day they were obtained from the ships tanker. Samples acidified with nitric acid, and kept for analysis.*
*Experimental features*	*Determine the concentration levels of metals including Zn, Al, Sr, and Co and metalloid of As in ballast water in commercial ships entering the Bushehr port.*
*Data source location*	*Bushehr harbor, Iran*
*Data accessibility*	*Data is with this article.*

**Value of the data**

• Data can be used as a base-line data for metals and metalloid contents in ballast water of commercial ships.
• Data shown here may motivate further studies on evaluate risk associated with ballast water discharge.
• Data show that ballast waters discharged by ship tankers in harbors are the main source of metal contamination for sea waters and coral reef in the discharge areas.
• Data confirmed stricter inspection and supervision as well as permanent monitory program (with respect to ballast water treatment) are necessary for management of ballast water in harbors.

## 1 Data

In the data, as shown in Table 1, the concentration levels of Zn, Al and Co of ballast water in commercial ships ranged from 1.23 to 6.58, 0.74 to 3.8 and 1.49 to 12.3 ppb respectively. The concentration levels of Sr was not detected (ND) in all examined samples. The concentration levels of As ranged from 0.11 to 1.84 ppb. The highest Zn concentration level was 6.58 ppb in sample S_6_ (Kuwait - Kuwait), whereas the lowest Zn concentration level was 1.23 ppb in sample S_17_ (Muscat- Oman). The highest and lowest concentration levels of Al were 3.8 and 0.74 ppb in samples S_34_ (Nagoya - Japan) and S_11_ (Kandla port - India) respectively. The highest and lowest content levels of Co were 12.3 and 1.49 ppb in samples S_22_ (Navlakhi - India) and S_26_ (Salalah - Oman) respectively. Finally, the highest concentration level of As was 1.84 ppb in sample S_22_ (Navlakhi - India) and the lowest level was 0.11 ppb in sample S_12_ (Ajman port - Emirate).

## 2 Experimental design, materials and methods

**Fig. 1:**
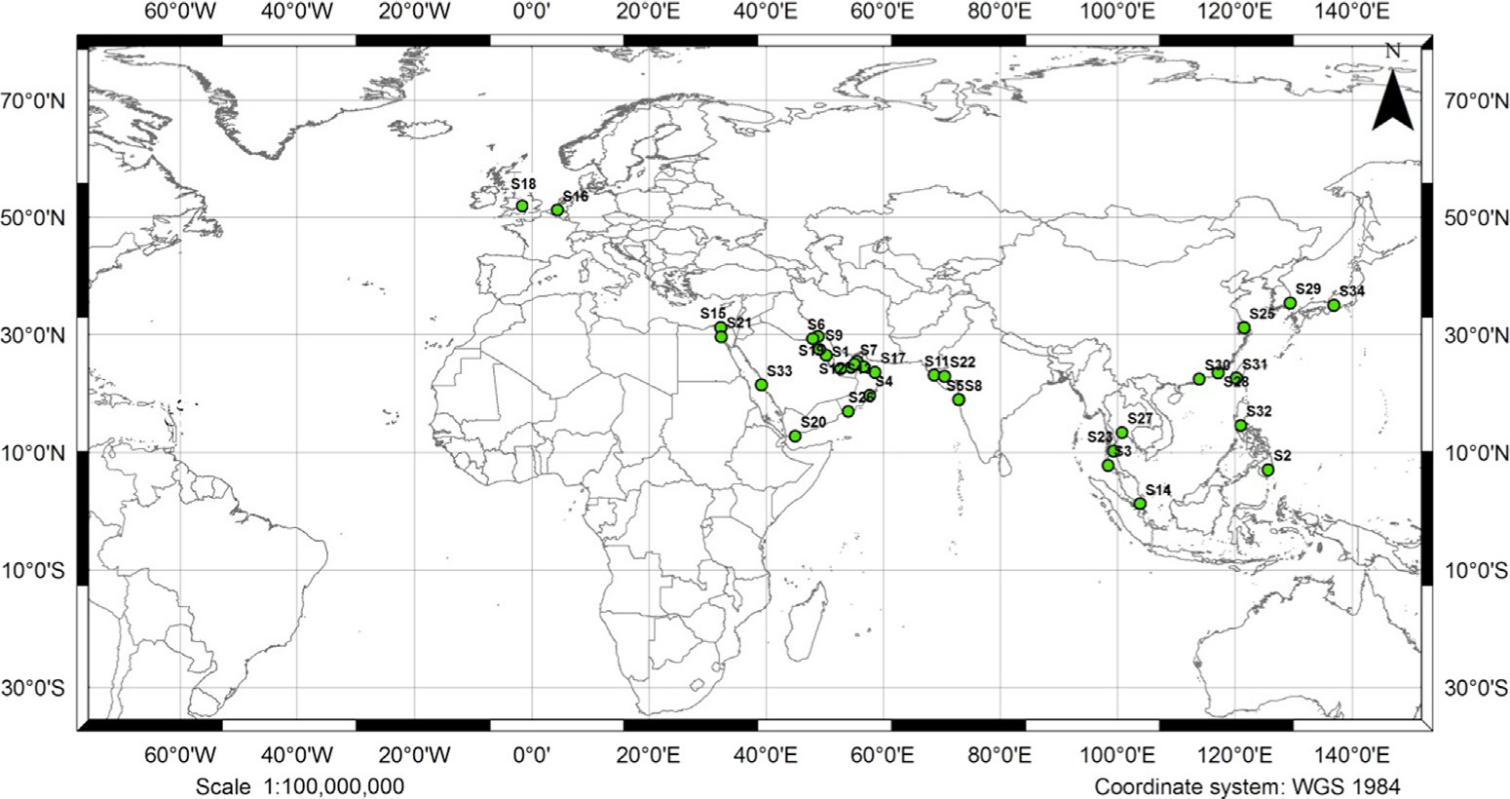
The geographical location of all ports that their ships arriving in the Bushehr port.

Ballast water samples were taken from commercial ships entering Bushehr port along the Persian Gulf during 15 February and 25 August 2016. Samples were from 34 different ports (see Fig. 1). Each sample was deposited in a 100 ml sterile container, placed in a cooler at −4 °C, and transported to the laboratory at the same day that they were obtained from ship tanker. Samples acidified with nitric acid, and kept for analysis. The concentrations levels of metals and metalloid were determined by using a graphite furnace absorption spectrometer (AAS) method [1] (Varian, SpectrAA 240, Australia).

## Figures and Tables

**Table 1:** Concentration levels (ppb) of metals (Zn, Al, Sr, and Co) and metalloid (As) in ballast water of commercial ships.

**Samples code**	**Location of harvesting ballast water**	**Zn**	**Al**	**Sr**	**Co**	**As**
S_1_	Dammam - Saudi Arabia	2.35	1.23	0	2.12	0.23
S_2_	Davao - Philippines	2.45	1.24	0	2.24	0.56
S_3_	Phuket - Thailand	6.33	1.56	0	3.21	0.41
S_4_	Duqm - Oman	2.36	0.87	0	6.53	0.26
S_5_	Jawaharlal Nehru Port - India	3.56	0.89	0	2.35	0.23
S_6_	Kuwait - Kuwait	6.58	2.31	0	4.29	0.34
S_7_	Jebel ali-Emirate	2.39	1.74	0	4.36	0.25
S_8_	Mumbai - India	2.47	1.68	0	5.14	1.01
S_9_	Shuwaikh - Kuwait	3.64	1.88	0	3.23	0.64
S_10_	Hamriya - Emirate	3.15	2.05	0	3.45	0.33
S_11_	Kandla port - India	2.09	0.74	0	2.56	0.64
S_12_	Ajman port - Emirate	3.47	1.93	0	2.87	0.11
S_13_	Mina rashid - Emirate	4	1.45	0	1.92	0.75
S_14_	Singapore - Singapore	1.97	1.68	0	4.34	0.64
S_15_	Port said - Egypt	2.36	1.37	0	3.17	0.96
S_16_	Antwerp- Belgium	2.88	0.96	0	5.26	1.09
S_17_	Muscat- Oman	1.23	1.23	0	3.31	1.23
S_18_	Portsmouth - U.K	3.33	1.29	0	3.56	1.23
S_19_	Basra - Iraq	2.35	1.78	0	4.56	0.45
S_20_	Aden - Yemen	3.21	1.56	0	8.96	0.56
S_21_	Suez - Egypt	5.12	2.11	0	8.69	1.56
S_22_	Navlakhi - India	6.35	2.23	0	12.3	1.84
S_23_	Bangkok - Thailand	3.15	3.11	0	3.39	0.34
S_24_	Sohar - Oman	2.94	2.59	0	3.15	0.36
S_25_	Shanghai - Chain	3.47	3.41	0	2.49	0.25
S_26_	Salalah – Oman	2.64	1.97	0	1.49	0.19
S_27_	Laem chabang - Thailand	3.35	1.8	0	4.11	0.48
S_28_	Hong kong - Chain	3.16	2.64	0	3.94	0.36
S_29_	Busan - South Korea	3.98	1.39	0	3.68	0.27
S_30_	Shenzhen - Chain	4.15	1.78	0	2.58	0.34
S_31_	Kaohsiung - Taiwan	3.27	2.41	0	3.47	0.26
S_32_	Manila - Philippines	1.67	2.31	0	4.61	0.34
S_33_	Jeddah - Saudi Arabia	3.74	3.12	0	4.39	0.18
S_34_	Nagoya - Japan	3.31	3.8	0	3.22	0.47
